# Predicting the Risk of Diabetic Foot Ulcers From Diabetics With Dysmetabolism: A Retrospective Clinical Trial

**DOI:** 10.3389/fendo.2022.929864

**Published:** 2022-07-12

**Authors:** Mingyang Jiang, Fu Gan, Meishe Gan, Huachu Deng, Xuxu Chen, Xintao Yuan, Danyi Huang, Siyi Liu, Baoyu Qin, Yanhong Wei, Shanggui Su, Zhandong Bo

**Affiliations:** ^1^ Department of Bone and Joint Surgery, The First Affiliated Hospital of Guangxi Medical University, Nanning, China; ^2^ Department of Urology Surgery, The Affiliated Hospital of Youjiang Medical University for Nationalities, Baise, China; ^3^ Department of Endocrinology, The People’s Hospital of Baise, Baise, China; ^4^ Department of Biochemistry and Molecular Biology, Basic Medical College, Guangxi Medical University, Nanning, China; ^5^ The Endocrine and Metabolic Disease area of Geriatrics, The First Affiliated Hospital of Guangxi Medical University, Nanning, China; ^6^ Department of Endocrinology, Wuming Hospital of Guangxi Medical University, Nanning, China

**Keywords:** diabetic foot ulcer (DFU), nomogram, risk factor, prediction model, dysmetabolism

## Abstract

**Background:**

Diabetic foot ulcer (DFU) in patients with type 2 diabetes mellitus (T2D) often leads to amputation. Early intervention to prevent DFU is urgently necessary. So far, there have been no studies on predictive models associated with DFU risk factors. Our study aimed to quantify the predictive risk value of DFU, promote health education, and further develop behavioral interventions to reduce the incidence of DFU.

**Methods:**

Data from 973 consecutive patients with T2D was collected from two hospitals. Patients from the Guangxi Medical University First Affiliated Hospital formed the training cohort (n = 853), and those from the Wuming Hospital of Guangxi Medical University formed the validation cohort (n = 120). Independent variable grouping analysis and multivariate logistic regression analysis were used to determine the risk factors of DFUs. The prediction model was established according to the related risk factors. In addition, the accuracy of the model was evaluated by specificity, sensitivity, predictive value, and predictive likelihood ratio.

**Results:**

In total, 369 of the 853 patients (43.3%) and 60 of the 120 (50.0%) were diagnosed with DFUs in the two hospitals. The factors associated with DFU were old age, male gender, lower body mass index (BMI), longer duration of diabetes, history of foot disease, cardiac insufficiency, no use of oral hypoglycemic agent (OHA), high white blood cell count, high platelet count, low hemoglobin level, low lymphocyte absolute value, and high postprandial blood glucose. After incorporating these 12 factors, the nomogram drawn achieved good concordance indexes of 0.89 [95% confidence interval (CI): 0.87 to 0.91] in the training cohort and 0.84 (95% CI: 0.77 to 0.91) in the validation cohort in predicting DFUs and had well-fitted calibration curves. Patients who had a nomogram score of ≥180 were considered to have a low risk of DFU, whereas those having ≥180 were at high risk.

**Conclusions:**

A nomogram was constructed by combining 12 identified risk factors of DFU. These 12 risk factors are easily available in hospitalized patients, so the prediction of DFU in hospitalized patients with T2D has potential clinical significance. The model provides a reliable prediction of the risk of DFU in patients with T2D.

## Introduction

Diabetes mellitus (DM) is a group of metabolic diseases characterized by long-term hyperglycemia (1), and the affected patients can develop multiple complications (2). Several characteristic pathological changes in the feet of patients with DM such as infection, diabetic foot ulcer (DFU), and neuroarthropathy are called diabetic foot syndrome (3). If diabetes is not controlled, then it can cause complications through complex metabolic pathways (4, 5): Peripheral neuropathy can lead to loss of sensation; peripheral artery disease may cause ischemia; a combination of both can eventually lead to foot ulcers (6, 7). Foot ulcers are a risk factor for foot infections, which greatly increases the probability of amputation (8). Therefore, among the vascular complications of diabetes, foot ulcer is the primary cause of hospitalization (8).

In patients with diabetes, DFU is one of the main causes of morbidity and mortality and a major public health problem, exerting a heavy burden on society (9). It has been reported that more than 20%–40% of medical resources related to diabetes are allocated to foot care (8). A foot ulcer is the most common manifestation of diabetic foot disease and has a very poor prognosis (10). The global prevalence of DFU is 6.3% [higher among males than females, type 2 (T2D) is higher than type 1 diabetes] (11), and DFU is the main cause of amputation in patients with diabetes (12). It is estimated that one diabetic foot is amputated every 20 s, with an annual mortality rate of 11% for DFUs and 22% for amputees (13). Ulcers appear in numb areas of the feet and legs, are often overlooked, can easily become infected, and eventually lead to amputation. Diabetic foot lesions are responsible for more hospitalizations than any other complication of diabetes (14). Other treatments have also been tried, such as skin grafting, vacuum sealing drainage, interventional therapy, and Tibetan transverse transport, but there are many complications in the operation, such as unhealed bone end, soft tissue infection, pain, and limb shortening deformity (15). Many patients have weak tolerance and lower compliance in the later period, which affects the effect of surgery.

Patients with diabetes are in a high glucose state for a long time, and their glucose metabolism is disordered, resulting in tissue hypoxia, an increase of damaging substances such as thromboxane, and complete vascular endothelial injury and promotion of microcirculation disorder (16). In addition, a high concentration of glucose glycosylates with protein and nucleic acid molecules inside and outside the blood vessel causes vascular cell dysfunction, promoting coagulation and thrombosis, leading to microangiopathy (17). As the mobility and phagocytosis of white blood cells (WBCs) are reduced, the immunity of the body is weakened due to peripheral neuropathy and vascular lesion. Small trauma can cause invasion and infection of microorganisms, so increased local oxygen consumption aggravates ischemia and gangrene occurs (18).

Prevention of DFU is better than treatment (19). Strengthening the management of patients at high risk of diabetic foot to ensure early detection, diagnosis, and treatment and to reduce the occurrence of DFUs can achieve twice the result with half the medical resources. Preventive measures of DFUs include regulating blood glucose levels, identifying and screening high-risk groups, patient education, and footwear management (20). Tight shoes, lack of foot care knowledge, and self-examination can cause repeated foot trauma, which becomes a trigger for DFUs, especially in the presence of peripheral neuropathy (21). However, there is limited evidence to support the preventive long-term benefit of patient education. A meta-analysis indicated that among all the recommended methods to prevent DFU, only foot temperature-guided avoidance therapy was effective in randomized controlled trials (22).

The clinical prediction model (also known as clinical prediction rules, prognosis models, or risk scores) refers to the use of multi-factor models to estimate the probability of a certain disease or its outcome in the future (23). The model includes the diagnostic and prognostic assessment. The prognostic model is concerned with the probability of recurrence, death, disability, and complications at a certain time in the future according to the current state of disease (23). Li et al. performed a predictive model to investigate diabetic retinopathy (DR) risk factors and predictive models by machine learning using a large sample dataset. They concluded that, with better comprehensive performance, the XGBoost model had high reliability to assess risk indicators of DR. An HbA1c value greater than 8%, nephropathy, a serum creatinine value greater than 100 µmol/L, insulin treatment, and diabetic lower extremity arterial disease were associated with an increased risk of DR (24). Zaidi et al. also conducted a multi−step ahead predictive model for blood glucose concentrations of patients with type 1 diabetes. They found that their model can capture the hyperglycemic and the hypoglycemic events better as compared to the ARX model but is slightly less accurate in the normoglycemic range values (25). Their studies all established predictive models associated with diabetes, but none described prognostic models associated with DFU. Our study is the first to predict DFU. In our study, by establishing a prognostic model, analyzing the risk factors of DFUs, and creating score tables and line charts, we aimed to provide more intuitive and powerful scientific tools for doctors and patients. We also aimed to quantify the risk value (probability) of DFUs in the future, promote health education, and advance behavioral interventions to reduce the incidence of DFUs.

## Methods

This was a hospital retrospective case-control study spanning from 1 January 2017 to 1 September 2019. The participants were patients with T2D who were treated at Guangxi Medical University First Affiliated Hospital and Wuming Hospital of Guangxi Medical University. All data were extracted from the hospital medical record system. The study was conducted following the Declaration of Helsinki (as revised in 2013). This study was approved by the Ethics Committees of Guangxi Medical University (No. 20220144), and individual consent for this retrospective analysis was waived. The patients did not receive financial compensation.

A total of 973 patients with T2D were identified. Because of the small number of patients with DFU in the validation group, propensity score matching (PSM) was applied to balance the number of the experimental group and the control group. The inclusion criteria were as follows: patients with type 2 diabetes with DFUs (the case group), and patients with type 2 diabetes without DFUs (the control group). The exclusion criteria were as follows: patients without T2D and foot breakage caused by trauma. Data of 429 patients in the case group were compared with that of 544 patients in the control group. Sociodemographic data, foot factors, diabetes-related risk factors, complications, and biochemical indicators were collected. The confidentiality of patient information was protected.

### Clinical Endpoint

The clinical variables of this study are shown in **Table 1**. Potential predictors are selected and included from published studies of diabetes (24, 25). Independent variable grouping analysis was used to determine the risk factors of DFUs: sociodemographic, foot factors, and biochemical indicators of diabetes. Sociodemographic data included gender, age, smoking history, alcohol use history, and body mass index (BMI). Age was confirmed by checking the patient’s ID number. Patients’ previous foot problems, such as non-diabetes–related foot ulcers, foot trauma, and/or foot surgery (such as surgical debridement, osteotomy, and fixation), were obtained through medical records or by self-report, without grouping. Diabetes risk factors retrieved from patients’ medical records, including course of the disease, family history, cardiac insufficiency, hypertension, hyperthyroidism, use of oral hypoglycemic agent (OHA), and use of insulin (INS). Diabetes-related complications included the following: peripheral vascular disease (PVD), peripheral neuropathy disease (PND), DR, and diabetic nephropathy; whereas biochemical indicators included the following: blood routine [red blood cell (RBC) count, WBC count, hemoglobin (Hb), platelet (PLT), neutrophil absolute value (NEUT), and lymphocyte absolute value (LY)], blood glucose {fasting (FBS), postprandial (PBS), glycosylated hemoglobin (HbA1c), urine glucose (GLU), blood lipids [total cholesterol (TCHOL), triglyceride (TG), high-density lipoprotein (HDLC), and low-density lipoprotein (LDLC)], renal function test [blood urea nitrogen (BUN) and serum creatinine (SCr)], and liver function test [total bilirubin (TBil), direct bilirubin (DBil), albumin (ALB), the ratio of glutamic oxaloacetic transaminase, glutamic pyruvic transaminase, and transaminase (ST/ALT)]. For the case group, these characteristics were determined on the basis of the data available in the patient’s medical history before the diagnosis of DFUs.

**Table 1:** Participant characteristics.

Variables	Training (n = 853)	Validation (n = 120)	P-value
Diabetic foot			0.302
Yes	369 (43.3)	60 (50.0)	
No	484 (56.7)	60 (50.0)	
Age, mean (SD), y	60.51 (12.7)	63.5 (10.4)	0.013
Gender			0.712
Male	590 (69.2)	81 (67.5)	
Female	263 (30.8)	39 (32.5)	
Duration, mean (SD), y	11.73 (6.9)	14.6 (11.9)	0.011
Vascular			0.122
Yes	441 (51.7)	53 (44.2)	
No	412 (48.3)	67 (55.8)	
Neuropathy			0.186
Yes	400 (46.9)	64 (53.3)	
No	453 (53.1)	56 (46.7)	
Retinopathy			0.613
Yes	210 (24.6)	27 (22.5)	
No	643 (75.4)	93 (77.5)	
Nephropathy			0.058
Yes	209 (24.5)	20 (16.7)	
No	644 (75.5)	100 (83.3)	
Cardiac			0.962
Yes	169 (19.8)	24 (20)	
No	684 (80.2)	96 (80)	
Hypertension			0.045
Yes	467 (54.7)	54 (45)	
No	386 (45.3)	66 (55)	
Hyperthyroidism			0.446
Yes	10 (1.2)	3 (2.5)	
No	843 (98.8)	117 (97.5)	
Course, y			<0.001
<10	490 (57.4)	95 (79.2)	
>10	363 (42.6)	25 (20.8)	
History			<0.001
Yes	76 (8.9)	28 (23.3)	
No	777 (91.1)	92 (76.7)	
Smoking			<0.001
Yes	319 (37.4)	15 (12.5)	
No	534 (62.6)	105 (87.5)	
Alcoholism			<0.001
Yes	316 (37.0)	11 (9.2)	
No	537 (63.0)	109 (90.8)	
Family			<0.001
Yes	190 (21.5)	1 (0.8)	
No	663 (75.1)	119 (99.2)	
BMI, kg/m^2^			<0.001
>24	378 (44.3)	30 (25.0)	
18.5–24	436 (51.1)	86 (71.7)	
<18.5	39 (4.6)	4 (3.3)	
WBC, ×10^9^/L			0.19
<10	651 (76.3)	85 (70.8)	
>10	202 (23.7)	35 (29.2)	
RBC, mean (SD), ×10^12^/L	4.3 (2.3)	4.5 (0.9)	0.457
Hb, mean (SD), mmol/L	117.8 (24.9)	121.5 (24.7)	0.126
PLT, ×10^9^/L			0.674
<300	552 (64.7)	80 (66.7)	
>300	301 (35.3)	40 (33.3)	
NEUT, ×10^9^/L			0.011
>7.5	172 (20.2)	36 (30.0)	
2–7.5	641 (75.1)	83 (69.2)	
<2	40 (4.7)	1 (0.8)	
LY, ×10^9^/L			0.589
>4	12 (1.4)	2 (1.7)	
0.8–4	796 (93.3)	109 (90.8)	
<0.8	45 (5.3)	9 (7.5)	
FBS, mmol/L			<0.001
≤6.1	244 (28.6)	14 (11.7)	
>6.1	609 (71.4)	106 (88.3)	
PBS, mmol/L			0.26
≤7.8	141 (16.5)	15 (12.5)	
>7.8	712 (83.5)	105 (87.5)	
HbA1c, %			0.407
≤6	60 (7.0)	6 (5.0)	
>6.1	793 (93.0)	114 (95.0)	
TCHOL, mmol/L			0.751
≤5.2	651 (76.3)	90 (75.0)	
>5.2	202 (23.7)	30 (25.0)	
TG, mmol/L			0.387
≤1.7	581 (68.1)	77 (64.2)	
>1.7	272 (31.9)	43 (35.8)	
HDLC, mmol/L			0.292
≤2	845 (99.1)	117 (97.5)	
>2	8 (0.9)	3 (2.5)	
LDLC, mmol/L			0.013
≤3.12	626 (73.4)	75 (62.5)	
>3.12	227 (26.6)	45 (37.5)	
BUN, mmol/L			0.016
≤7.1	568 (66.6)	93 (77.5)	
>7.1	285 (33.4)	27 (22.5)	
SCr, μmol/L			0.236
≤133	710 (83.2)	105 (87.5)	
>133	143 (16.8)	15 (12.5)	
TBil, μmol/L			0.009
≤17.1	781 (91.6)	118 (98.3)	
>17.1	72 (8.4)	2 (1.7)	
DBil, μmol/L			0.099
≤7	807 (94.6)	109 (90.8)	
>7	46 (5.4)	11 (9.2)	
ALB, g/L			0.011
≤51	842 (98.7)	114 (95.0)	
>51	11 (1.3)	6 (5.0)	
AST, U/L			0.673
≤40	771 (90.4)	107 (89.2)	
>40	82 (9.6)	13 (10.8)	
ALT, U/L			0.024
≤40	752 (88.2)	97 (80.8)	
>40	101 (11.8)	23 (19.2)	
GLU			0.001
−	405 (47.5)	37 (30.8)	
+	448 (52.5)	83 (69.2)	
OHA			0.064
Yes	572 (67.1)	70 (58.3)	
No	281 (32.9)	50 (41.7)	
INS			0.004
Yes	542 (63.5)	92 (72.7)	
No	311 (36.5)	28 (23.3)	
AST/ALT			<0.001
<1	380 (44.5)	107 (89.2)	
≥1	473 (55.5)	13 (10.8)	

ALT, alanine aminotransferase; ALB, albumin; AST, aspartate aminotransferase; BMI, body mass index; cardiac, cardiac insufficiency; BUN, blood urea nitrogen; DBil, direct bilirubin; FBS, fasting blood glucose; GLU, urine glucose; Hb, hemoglobin; HbA1c, glycosylated hemoglobin; HDLC, high-density lipoprotein; INS, insulin; LDLC, low-density lipoprotein; LY, lymphocyte absolute value; NEUT, neutrophil absolute value; OHA, oral hypoglycemic agent; PBS, postprandial blood glucose; PLT, platelet; RBC, red blood cell; SCr, serum creatinine; TBil, total bilirubin; TCHOL, total cholesterol blood lipids; TG, triglyceride; WBC, white blood cell; y, year; SD, standard deviation.

### Statistical Analysis

The ratio of the case to control participants was approximately 1:1. All data were encoded and analyzed using SPSS 25.0 (IBM Corp., Armonk, IL, USA) and R 3.6.1 (The R Foundation for Statistical Computing, Vienna, Austria). A chi-square test was used to check the statistical independence between the two groups. Univariate logistic regression analysis was used to evaluate the importance of each variable in the training cohort, to investigate the independent risk factors of DFUs. All significant levels of variables associated with DFUs were analyzed by multivariate logistic regression analysis. A line diagram was made using the rms software package (http://www.r-project.org/) of R 3.6.1, based on the results of multiple logistic regression analysis. The line chart was based on the proportional conversion of each regression coefficient in a multivariate logistic regression on a scale of 0 to 100 points. The influence of the variable with the highest β coefficient (absolute value) was assigned 100 points. The total points were obtained by combining these points with independent variables and were then converted into prediction probability. The predictive performance of the line chart was measured using the consistency index (C index) and calibrated with 1,000 bootstrapped samples to reduce the over-fitting deviation.

The clinical use of the model was based on the line chart to calculate the total score of each patient. The optimal critical value was calculated by analyzing the receiver operating characteristic (ROC) curve, which was determined by maximizing the Youden index. The accuracy of the optimal critical value was evaluated by specificity, sensitivity, predictive value, and predictive likelihood ratio. The acceptable threshold values of ROC, sensitivity, and specificity of the predictive model were 0.8. A two-sided P-value <0.05 was considered statistically significant (26).

## Results

### Clinical Characteristics

During the study period, 1,288 patients with T2D were selected according to the inclusion criteria. The training cohort had 853 patients (369 in the case and 484 in the control group). The validation cohort had 435 patients, from which 120 (60 each, for the case and the control group) were selected according to PSM. **Table 1** lists the clinical characteristics of the patients. The baseline clinical data of the training cohorts were similar to that of the validation cohort.

### Nomogram Development and Validation

All data for the study were extracted from medical records. Univariate logistic analysis was performed to exclude significantly unrelated variables, as shown in **Table 2**. The results of the multivariate analysis were reported as odds ratio [95% confidence interval (CI)] as shown in **Table 3**.

**Table 2:** Univariate logistic regression analysis of DFU based on preoperative data in the training cohort.

Variable	OR (95% CI)	P-value
Age	1.03 (1.02–1.04)	<0.001
Gender (male vs. female)	1.90 (1.40–2.58)	<0.001
Duration	1.16 (1.13–1.20)	<0.001
Vascular (yes vs. no)	0.24 (0.18–0.32)	<0.001
Neuropathy (yes vs. no)	0.37 (0.28–0.49)	<0.001
Retinopathy (yes vs. no)	0.58 (0.41–0.79)	<0.001
Nephropathy (yes vs. no)	1.49 (1.09–2.04)	0.01
Cardiac (yes vs. no)	1.76 (1.25–2.47)	0.001
Hypertension (yes vs. no)	1.14 (0.87–1.50)	0.33
Hyperthyroidism	0.32 (0.05–1.30)	0.16
Course, y	1.83 (1.38–2.43)	<0.001
History	8.25 (4.54–16.30)	<0.001
Smoking	1.04 (0.79–1.38)	0.77
Alcoholism	0.91 (0.69–1.21)	0.53
Family	0.45 (0.32–0.64)	<0.001
OHA	3.78 (2.79–5.10)	<0.001
INS	0.61 (0.46–0.81)	<0.001
BMI, kg/m^2^	1.82 (1.37–2.42)	<0.001
	3.82 (1.93–7.90)	<0.001
WBC, ×10^9^/L	5.00 (3.55–7.13)	<0.001
RBC, mean (SD), ×10^12^/L	0.30 (0.25–0.37)	<0.001
Hb, mean (SD), mmol/L	0.95 (0.94–0.96)	<0.001
PLT, ×10^9^/L	1.57 (3.57–6.54)	<0.001
NEUT, ×10^9^/L	0.19 (0.13–0.27)	<0.001
	0.03 (0.01–0.08)	<0.001
LY, ×10^9^/L	7.99 (1.54–146.47)	0.05
	30.2 (5.09–583.80)	0.002
FBS, mmol/L	1.56 (1.15–2.12)	0.005
PBS, mmol/L	2.46 (1.66–3.71)	<0.001
HbA1c, %	0.56 (0.33–0.95)	0.03
TCHOL, mmol/L	0.38 (0.26–0.53)	<0.001
TG, mmol/L	0.44 (0.32–0.60)	<0.001
HDLC, mmol/L	0.43 (0.06–1.90)	0.31
LDLC, mmol/L	0.41 (0.30–0.57)	<0.001
BUN, mmol/L	2.51 (1.88–3.36)	<0.001
SCr, μmol/L	3.15 (2.17–4.63)	<0.001
TBil, μmol/L	0.63 (0.37–1.04)	0.08
DBil, μmol/L	1.76 (0.97–3.24)	0.06
AST/ALT	0.81 (0.61–1.06)	0.13
GLU	1.22 (0.93–1.60)	0.16

DFU, diabetic foot ulcer; ALT, alanine aminotransferase; ALB, albumin; AST, aspartate aminotransferase; BMI, body mass index; BUN, blood urea nitrogen; cardiac, cardiac insufficiency; FBS, fasting blood glucose; GLU, urine glucose; Hb, hemoglobin; HbA1c, glycosylated hemoglobin; HDLC, high-density lipoprotein; INS, insulin; LDLC, low-density lipoprotein; LY, lymphocyte absolute value; NEUT, neutrophil absolute value; OHA, oral hypoglycemic agent; PBS, postprandial blood glucose; PLT, platelet; RBC, red blood cell; SCr, serum creatinine; TBil, total bilirubin; DBil, direct bilirubin; TCHOL, total cholesterol blood lipids; TG, triglyceride; WBC, white blood cell; y, year; OR, odd ratio; CI, confidence interval.

**Table 3:** Multivariate logistic regression analysis of DFU based on preoperative data in the training cohort.

Variable	β^a^	OR (95% CI)	P-value
Age	0.02	1.02 (1.00–1.04)	0.05
Gender (male vs. female)	1.22	3.39 (1.86–6.27)	<0.001
Duration	0.10	1.10 (1.05–1.15)	<0.001
Vascular (yes vs. no)	−1.39	0.25 (0.15–0.40)	<0.001
Neuropathy (yes vs. no)	−0.62	0.54 (0.33–0.89)	0.02
Retinopathy (yes vs. no)	0.02	1.02 (0.57–1.81)	0.95
Nephropathy (yes vs. no)	0.25	1.28 (0.73–2.25)	0.39
Cardiac (yes vs. no)	0.64	1.90 (1.08–3.36)	0.03
Hypertension (yes vs. no)	0.08	1.08 (0.66–1.80)	0.75
Hyperthyroidism (yes vs. no)	−1.17	0.31 (0.03–2.14)	0.27
Course, y	0.30	1.35 (0.83–2.20)	0.23
History (yes vs. no)	1.94	6.95 (2.91–17.62)	<0.001
Smoking (yes vs. no)	−0.27	0.76 (0.40–1.43)	0.39
Alcoholism (yes vs. no)	−0.11	0.90 (0.48–1.68)	0.73
Family (yes vs. no)	−0.58	0.56 (0.31–1.00)	0.05
OHA (no vs. yes)	−0.54	0.58 (0.35–0.96)	0.03
INS (no vs. yes)	0.00	1.00 (0.61–1.66)	0.99
BMI, kg/m^2^ (>24 vs. 18.5–24)	0.00	1.00 (0.61–1.61)	0.99
BMI, kg/m^2^ (18.5–24 vs.<18.5)	1.31	3.72 (1.05–13.68)	0.04
RBC,×10^12^/L	−0.24	0.79 (0.56–1.00)	0.12
WBC, ×10^9^/L (>10 vs. ≤10)	1.09	2.96 (1.19–7.40)	0.02
Hb, mmol/L	−0.03	0.97 (0.96–0.99)	<0.001
PLT, ×10^9^/L (>300 vs. ≤300)	0.86	2.36 (1.38–4.06)	0.002
NEUT, ×10^9^/L (>7.5 vs. 2–7.5)	0.06	1.06 (0.39–2.88)	0.9
NEUT, ×10^9^/L (2–7.5 vs. <2)	−1.90	0.15 (0.018–0.77)	0.04
LY, ×10^9^/L (>4 vs. 0.8–4)	2.89	18.0 (2.13–402.23)	0.02
LY, ×10^9^/L (0.8–4 vs. <0.8)	3.32	27.6 (2.43–722.18)	0.01
FBS, mmol/L (>6.1 vs. ≤6.1)	0.30	1.35 (0.80–2.32)	0.27
PBS, mmol/L (>7.8 vs. ≤7.8)	0.74	2.10 (1.08–4.18)	0.03
HbA1c, % (>6 vs. ≤6)	−0.30	0.74 (0.30–1.82)	0.51
TCHOL, mmol/L (>5.2 vs. ≤5.2)	−0.14	0.87 (0.39–1.95)	0.74
TG, mmol/L (>1.7 vs. ≤1.7)	−0.46	0.63 (0.38–1.06)	0.08
HDLC, mmol/L (>2 vs. ≤2)	1.05	2.85 (0.14–20.82)	0.37
LDLC, mmol/L (>3.12 vs. ≤3.12)	−0.30	0.74 (0.35–1.56)	0.42
BUN, mmol/L (>7.1 vs. ≤7.1)	0.15	1.16 (0.66–2.02)	0.61
SCr, μmol/L (>133 vs. ≤133)	−0.29	0.75 (0.36–1.53)	0.42
TBil, μmol/L (>17.1 vs. ≤17.1)	−0.33	0.72 (0.21–2.11)	0.57
DBil, μmol/L (>7 vs. ≤7)	0.18	1.20 (0.27–5.40)	0.81
GLU (+ vs. −)	0.33	1.39 (0.87–2.21)	0.17
AST/ALT (<1 vs. ≥1)	0.12	1.13 (0.72–1.78)	0.59

β^a^, regression coefficient. DFU, diabetic foot ulcer; ALT, alanine aminotransferase; ALB, albumin; AST, aspartate aminotransferase; BMI, body mass index; BUN, blood urea nitrogen; cardiac, cardiac insufficiency; FBS, fasting blood glucose; GLU, urine glucose; Hb, hemoglobin; HbA1c, glycosylated hemoglobin; HDLC, high-density lipoprotein; INS, insulin; LDLC, low-density lipoprotein; LY, lymphocyte absolute value; NEUT, neutrophil absolute value; OHA, oral hypoglycemic agent; PBS, postprandial blood glucose; PLT, platelet; RBC, red blood cell; SCr, serum creatinine; TBil, total bilirubin; DBil, direct bilirubin; TCHOL, total cholesterol blood lipids; TG, triglyceride; WBC, white blood cell; y, year; OR, odd ratio; CI, confidence interval.

**Figure 1:**
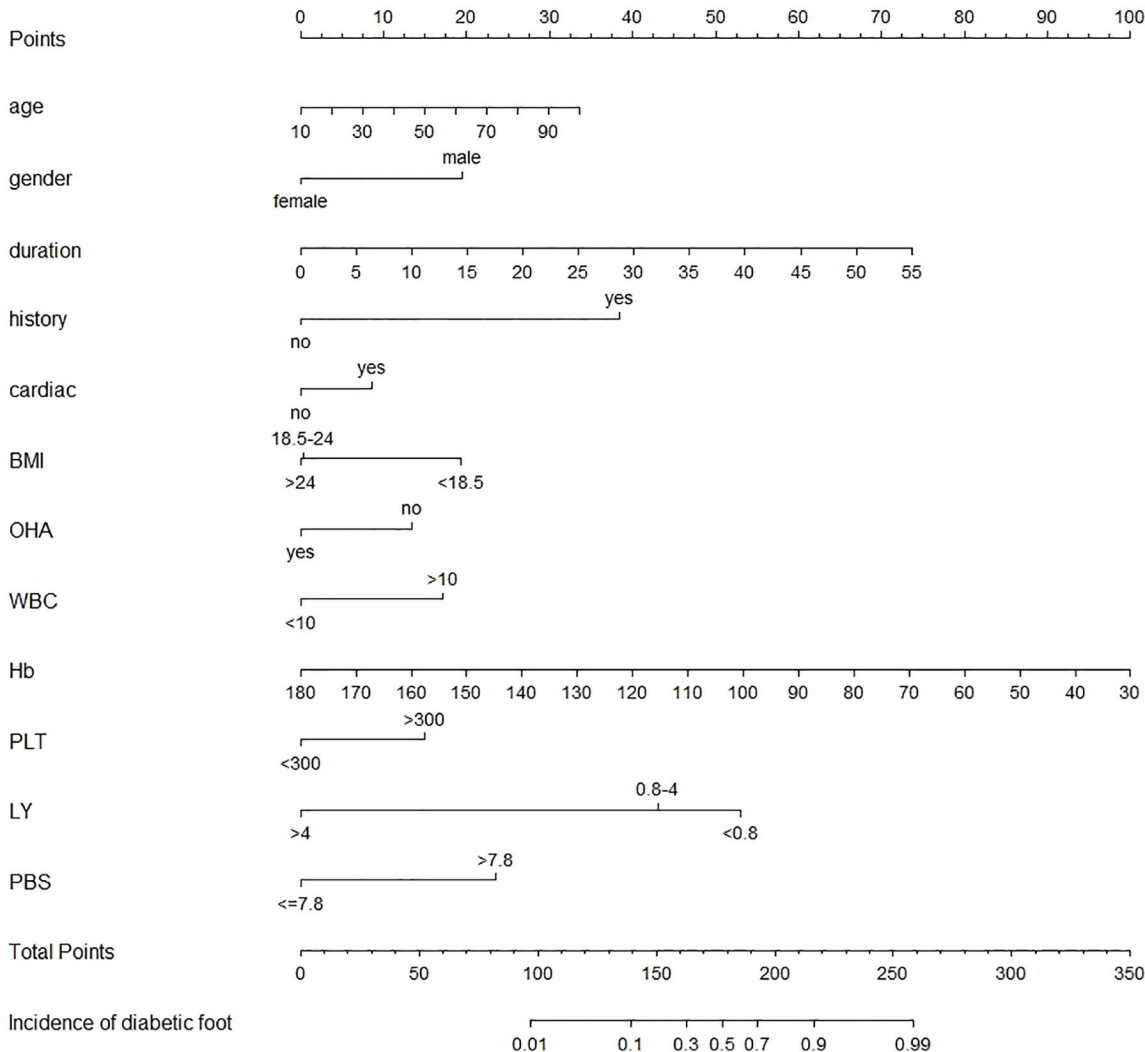
Nomogram to estimate the risk of DFU in patients with diabetes. To use the nomogram, find the position of each variable on the corresponding axis, draw a line to the points axis for the number of points, add the points from all variables, and draw a line from the total point axis to determine the DFU probabilities at the lower line of the nomogram. cardiac, cardiac insufficiency; DFU, diabetic foot ulcer; BMI, body mass index; Hb, hemoglobin; LY, lymphocyte absolute value; OHA, oral hypoglycemic agent; PBS, postprandial blood glucose; PLT, platelet; WBC, white blood cell.

**Figure 2:**
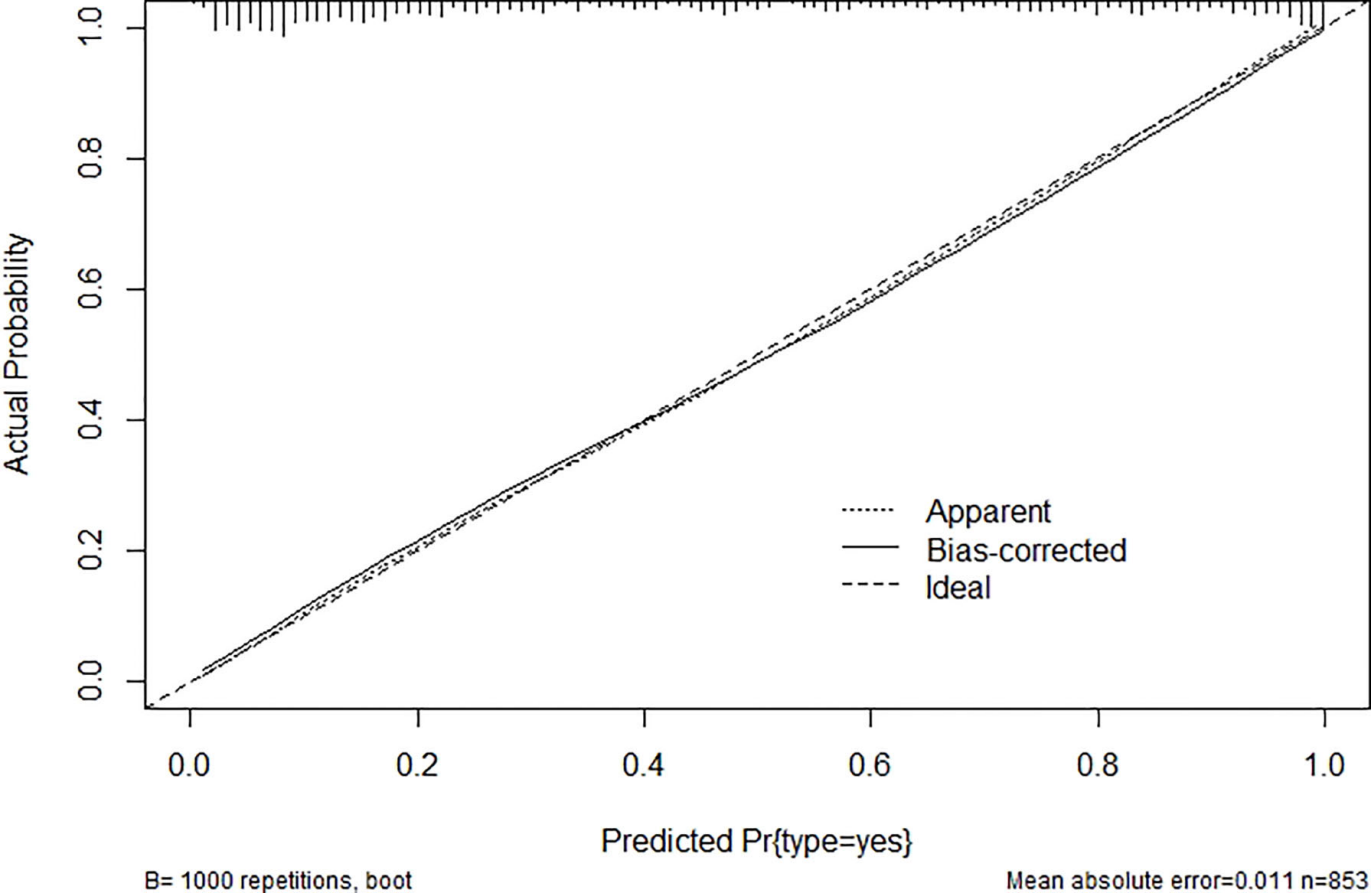
Area under ROC curve (concordance index) **(A)** in the training cohort and **(B)** in the validation cohort. ROC, receiver operating characteristic.

**Figure 3:**
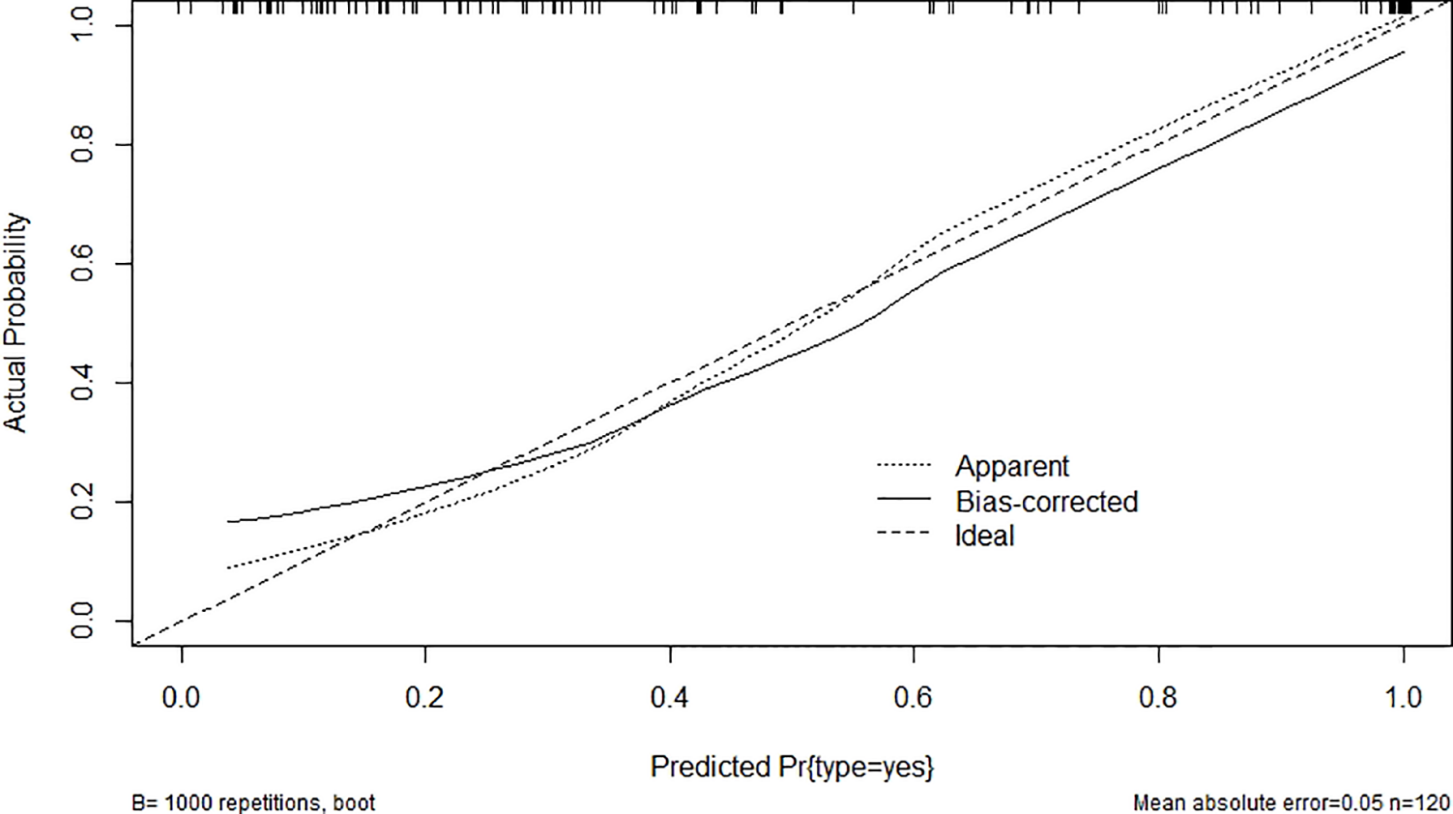
Validity of the predictive performance of the nomogram in estimating the risk of DFU: **(A)** in the training cohort (n = 853) and **(B)** in the validation cohort (n = 120). DFU, diabetic foot ulcer.

After modification, 12 independently related risk factors (age, gender, duration, cardiac insufficiency, history of foot diseases, family history, BMI, WBC, PLT, LY, PBS, and use of OHA) were used to form a DFU risk estimation nomogram (**Figure 1**), which demonstrated good accuracy with a C index of 0.89 (95% CI: 0.87 to 0.91) (**Figure 2A**) in the training cohort. In addition, the nomogram displayed a C index of 0.84 (95% CI: 0.77 to 0.91) (**Figure 2B**) in the validation cohort. In addition, the calibration chart was shown on the graph, and the risk estimation of the line chart fit well (**Figure 3A**) in the training cohort, whereas in the validation cohort, the risk estimation also showed a good calibration curve (**Figure 3B**).

### Risk of DFUs Based on the Nomogram Scores

The optimal cutoff value for the nomogram was determined to be 180. The sensitivity, specificity, positive, and negative predictive values used in differentiating the presence of DFUs were 81.1%, 81.1%, 73.4%, and 87.0%, respectively, in the training cohort, and 80.0%, 75.4%, 73.3%, and 81.7%, respectively, in the validation cohort (**Table 4**).

**Table 4:** Accuracy of the prediction score of the nomogram for estimating the risk of DFU.

Variable	Value (95% CI)	
	Training cohort	Validation cohort
Area under ROC curve, concordance index	0.89 (0.87–0.91)	0.84 (0.77–0.91)
Cutoff score	180	180
Sensitivity, %	81.1 (76.4–85.1)	80.0 (66.6–89.1)
Specificity, %	81.1 (77.4–84.3)	75.4 (62.9–84.9)
Positive predictive value, %	73.4 (68.6–77.8)	73.3 (60.1–83.5)
Negative predictive value, %	87.0 (83.5–89.8)	81.7 (69.1–90.1)
Positive likelihood ratio	4.29 (3.56–5.16)	3.25 (2.08–5.07)
Negative likelihood ratio	0.23 (0.19–0.29)	0.27 (0.15–0.45)

DFU, diabetic foot ulcer; ROC, receiver operating characteristic; CI, confidence interval.

## Discussion

As a serious diabetic complication, DFUs seriously affect the quality of life of patients with T2D (27). The lifetime incidence of foot ulcers is estimated to be 15%–25% among patients with diabetes (28); incorporating additional data, 19%–34% of patients are likely to be affected. Peripheral artery disease is an important risk factor for the development of DFU (29). However, the diagnosis of DFU in diabetes is challenging due to neuropathy and arterial calcification (30). The commonly used bedside tests are either insensitive or have little supporting evidence for their use. There is a good correlation between duplex ultrasound and angiography, but a full scan is difficult to fathom and time-consuming to perform (31). None of the existing methods can predict early-stage DFUs, and patients are often at risk of amputation when symptoms develop. A nomogram has good discrimination characteristics, has high accuracy in the prediction of results among the available prediction tools, and is easy to use. Our proposed nomogram incorporated comprehensive and easily available preoperative variables performed well (C index values of 0.89 in the training and 0.84 in the validation cohorts), and the optimal calibration curves demonstrated agreements between actual observation and prediction (26).

A meta-analysis published in 2019 showed that risk factors for DFUs include male gender, smoking, duration of past DFUs, plantar ulcers, peripheral artery disease, and diabetic peripheral neuropathy. There were no significant differences in age, BMI, total cholesterol, DR, diabetic nephropathy, or hypertension (32). Nanwani et al. (33) suggested that crucial risk factors include male gender, smoking, hyperlipidemia, hypertension, cardiac history, and the co-occurrence of diabetic nephropathy and DR. However, literature has shown that, due to too many risk factors, their interaction, and different baseline patient characteristics, the conclusions drawn from studies around the world are controversial (24, 25). Our study suggested that older age, male gender, lower BMI, longer duration of diabetes, history of foot disease, cardiac insufficiency, no use of OHA, higher WBC count, higher PLT count, lower Hb concentration, lower LY absolute value, and higher PBS were significantly associated with high incidence rates of DFUs. Our results were somewhat consistent with previous studies (34), but there were also great differences. For example, the research of Zubair et al. (34) stated that there is a positive association with gender, diabetes duration, ulcer size, grade of ulcer, amputation rate, hospital stay, Hb, SGOT/AST, and triglyceride. However, our study showed that lower BMI (especially BMI <18.5) is related to a higher risk of developing DFUs, which may be due to thinner patients having had a longer course of the disease. Factors such as smoking, diabetic nephropathy, and DR, which have been linked to the occurrence of DFU, were excluded from the final model due to the insignificant statistical test (P > 0.05) in the multivariate logistic regression analysis. PVD and PNDs were recognized as significant signs for DFUs (35); but in our study, multivariate logistic regression analysis showed that the DFUs group had a lower risk of the two symptoms, which was a challenge to the previous findings. We postulate that this was because the conditions were common complications in T2D, with or without DFU. Because this was a retrospective study, it was not possible to determine whether the occurrence of PVD and PND was the cause of DFUs, but there was a causal relationship, so more far-reaching prospective studies are needed. The goal of the clinical prediction model was to warn patients with diabetes, with risk factors but without foot symptoms, about early prevention. If they already had PVD or PND, then imaging methods such as angiography were used for visualization, and the patient was promptly treated.

For patients with diabetes mellitus with high scores, we have the following suggestions to avoid DFU (36): (I) Pay attention to improving circulation: to prevent the affected part from being compressed, and pay attention to frequently turning over during lying to reduce the time of local compression, and use stents when necessary. (II) Foot movement: patients must pay attention to it when taking each step. It is best that all toes and forepaw consciously and actively exert force, especially the big toe so that the foot arch can participate in exerting force and effectively exercise the muscles of the foot; the heel almost does not touch the ground or just touches the ground lightly, which allows the foot arch to bear most of the weight and increases the weight-bearing efficiency of the foot. (III) Effective control of blood glucose: Good control of blood glucose is the most beneficial measure to reduce complications of diabetes, and control of glycated hemoglobin within the normal range can reduce the occurrence of complications. (IV) Actively prevent foot trauma: form the habit of checking the foot every day; quit smoking; soak your feet in warm water for a limited period each day. Trim toenails correctly and select footwear appropriately; do not walk barefoot, barefoot shoes; keep skin clean and moist, and prevent dry itching and scratches.

This is the first early clinical predictive model of DFU. Previous articles, in recent decades, have been limited to identifying foot risk factors in patients with diabetes, which are interrelated; separate, single, or multiple factors cannot facilitate accurate diagnosis. In this study, by collecting data from nearly a thousand patients and integrating significant risk factors, we made a predictive scoring model. Those with high scores can take early DFU preventive measures, such as foot care, greatly reducing the incidence of amputation and related financial burden.

There were limitations to this study: (I) The reliability of the nomogram was not confirmed and requires confirmation *via* prospective studies. (II) This analysis was based on the data of two single institutions in Guangxi, China. It is necessary to verify the results through comparison with those of other centers. (III) Although the nomogram had good predictive accuracy (cutoff point of 180), there were still false positives and false negatives in the training and validation cohorts. (IV) Using the nomogram to estimate the risk of secondary DFUs in patients with diabetes to guide clinical treatment is a new concept. Diabetes has other complications and other factors that were not included in the model, such as the general performance of patients and the functional reserve of the liver, which should also be considered in the future. If major clinical decisions are to be made, then larger sample sizes are needed for more in-depth research. (V) The model was based on clinical data, and the use of angiography and other imaging techniques may further improve accuracy, but this needs the cooperation of patients and financial support.

## Conclusions

A nomogram was constructed by combining 12 risk factors of DFU. The model provides a reliable prediction of the risk of DFUs in patients with T2D.

## Data Availability Statement

The original contributions presented in the study are included in the article/Supplementary Material. Further inquiries can be directed to the corresponding authors.

## Ethics Statement

This study was approved by the Ethics Committees of Guangxi medical university (No. 20220144), and individual consent for this retrospective analysis was waived.

## Author Contributions

Conception and design: MJ, FG, and MG; Administrative support: ZB and SS; Provision of study materials or patients: BQ and YW; Collection and assembly of data: HD, XY, DH, XC, and SL; Data analysis and interpretation: MJ, FG, and MG; Manuscript writing: All authors; Final approval of manuscript: All authors.

## Conflict of Interest

The authors declare that the research was conducted in the absence of any commercial or financial relationships that could be construed as a potential conflict of interest.

## Publisher’s Note

All claims expressed in this article are solely those of the authors and do not necessarily represent those of their affiliated organizations, or those of the publisher, the editors and the reviewers. Any product that may be evaluated in this article, or claim that may be made by its manufacturer, is not guaranteed or endorsed by the publisher.
